# Real-World Health Care Outcomes and Costs Among Patients With Juvenile Idiopathic Arthritis in Spain

**DOI:** 10.36469/001c.85088

**Published:** 2023-12-20

**Authors:** Jordi Antón, Estefania Moreno Ruzafa, Mireia Lopez Corbeto, Rosa Bou, Judith Sánchez Manubens, Sonia Carriquí Arenas, Joan Calzada Hernández, Violetta Bittermann, Carolina Estepa Guillén, Juan Mosquera Angarita, Lucía Rodríguez Díez, Estíbaliz Iglesias, Miguel Marti Masanet, Berta Lopez Montesinos, Maria I. González Fernández, Alfonso de Lossada, Carmen Peral, Mónica Valderrama, Noelia Llevat, María Montoro Álvarez, Immaculada Calvo Penadés

**Affiliations:** 1 Pediatric Rheumatology Department Hospital San Joan de Déu, Barcelona, Spain; 2 Universitat Autònoma de Barcelona, Barcelona, Spain; 3 Pediatric Rheumatology Section, Vall d’Hebron Barcelona Hospital Campus, Barcelona, Spain; 4 Servei de Pediatria, Hospital Parc Taulí Sabadell; 5 Pediatric Rheumatology Unit, Hospital Universitario y Politécnico La Fe, Valencia, Spain; 6 Pediatric Rheumatology Unit, Hospital Universitario y Politécnico La Fe, Valencia, Spain.; 7 Pfizer SLU, Alcobendas-Madrid, Spain

**Keywords:** juvenile arthritis, healthcare resources, family impact, direct cost, indirect cost

## Abstract

**Background:** Juvenile idiopathic arthritis (JIA) is the most frequent chronic rheumatic disease in children. If inflammation is not adequately treated, joint damage, long-term disability, and active disease during adulthood can occur. Identifying and implementing early and adequate therapy are critical for improving clinical outcomes. The burden of JIA on affected children, their families, and the healthcare system in Spain has not been adequately assessed. The greatest contribution to direct costs is medication, but other expenses contribute to the consumption of resources, negatively impacting healthcare cost and the economic conditions of affected families.

**Objective:** To assess the direct healthcare, indirect resource utilization, and associated cost of moderate-to-severe JIA in children in routine clinical practice in Spain.

**Methods:** Children were enrolled in this 24-month observational, multicentric, cross-sectional, retrospective study (N = 107) if they had been treated with biologic disease-modifying anti-rheumatic drugs (bDMARDs), had participated in a previous study (ITACA), and continued to be followed up at pediatric rheumatology units at 3 tertiary Spanish hospitals. Direct costs included medication, specialist and primary care visits, hospitalizations, emergency visits or consultations, surgeries, physiotherapy, and tests. Indirect costs included hospital travel expenses and loss of caregiver working hours. Unitary costs were obtained from official sources (€, 2020).

**Results:** Overall, children had inactive disease/low disease activity according to JADAS-71 score and very low functional disability as measured by Childhood Health Assessment Questionnaire score. Up to 94.4% of children received treatment, mainly with bDMARDs as monotherapy (84.5%). Among anti-TNFα treatments, adalimumab (47.4%) and etanercept (40.2%) were used in similar proportions. Annual mean (SD) total JIA cost was €7516.40 (€5627.30). Average cost of pharmacological treatment was €3021.80 (€3956.20), mainly due to biologic therapy €2789.00 (€3399.80). Direct annual cost (excluding treatments) was €3654.60 (€3899.00). Indirect JIA cost per family was €747.20 (€1452.80).

**Conclusion:** JIA causes significant costs to the Spanish healthcare system and affected families. Public costs are partly due to the high cost of biologic treatments, which nevertheless remain an effective long-term treatment, maintaining inactive disease/low disease activity state; a very low functional disability score; and a good quality of life.

## BACKGROUND

Juvenile idiopathic arthritis (JIA) represents a varied group of conditions characterized by chronic inflammatory arthritis that occurs in children younger than 16 years of age and persists for more than 6 weeks.^1^ JIA is the most frequent chronic rheumatic disease in children, with an annual incidence of 2 to 20 per 100 000 children^2–4^ and a prevalence in Europe of 16.9 to 167 per 100 000.^5–7^ In Spain, a JIA prevalence of 39.7 to 44.8 per 100 000 has been described in Catalonia^8^ and 51.4 children and adolescents under 16 years of age in Asturias.^9^ A recent study in Germany based on claims data analyses estimates a prevalence of JIA of approximately 0.10% (0.07%-0.10%) of individuals under 18 years of age.^10^ Symptoms of JIA vary depending on the type, but all forms share persistent joint pain, swelling, warmth, and stiffness. Besides joint problems, the inflammation associated with JIA can cause other symptoms, such as uveitis, rash, fever, and fatigue. If chronic, persistent joint inflammation is not adequately treated, permanent joint damage can occur^11–13^; it can also be associated with abnormal growth and, in some cases, long-term disability.^14^ Estimates indicate that more than 50% of patients will reach adulthood with active disease.^14^

Early appropriate identification and application of therapy are critical for improving clinical outcomes.^15^ Treatments available for JIA include nonsteroidal anti-inflammatory agents, intra-articular corticosteroid injections, conventional synthetic disease-modifying anti-rheumatic drugs (csDMARDs) and biologic disease-modifying anti-rheumatic drugs (bDMARDs), including tumor necrosis factor inhibitors (anti-TNF), and other mechanisms of action (L-1 receptor antagonists, IL-6 receptor antagonists, anti–T cell–specific inhibitors). Recently tofacitinib, a JAK inhibitor, has been approved for patients with JIA.^16^ Recommendations for the treatment of JIA include starting treatment with methotrexate as soon as possible and, for children with risk factors, bDMARDs could be considered as initial treatment. If disease activity is moderate or high with methotrexate (MTX), starting bDMARDs is the most acceptable step.

As a chronic condition, JIA places a significant burden on affected children and their families^14,17^ and on the healthcare system.^14^ The largest contribution to direct costs is medication,^12,18,19^ mainly biologic therapies, which may cost 10 times more than conventional therapies.^20^ Among the bDMARDs, the most experience has been with the anti-TNF therapies, which have been used for more than 20 years since their approval. However, data regarding the cost of anti-TNF drugs in JIA are scarce.^12,14,21^ The cost difference in csDMARD and anti-TNF treatment, with anti-TNF treatment being more expensive, and the lack of long-term cost-effectiveness data on anti-TNF treatment, have contributed to anti-TNFs being prescribed when csDMARDs have failed. When the drug cost itself is excluded, direct and indirect healthcare costs could be reduced in children treated with anti-TNF drugs.^12,19^

A child’s chronic illness is not limited to the child alone, as it affects all members of the family. Families with children affected by JIA can experience many difficulties, mainly related to the emotional and economic impact of the disease.^22^ Numerous studies have reported the burdens of caring for a child with JIA.^23–25^ The caregiver’s role can have an influence on employment in several respects, such as job resignation, absenteeism, and reduced productivity.^25^

General health care of children in Spain is most frequently provided by pediatricians, although from 14 to 18 years, follow-up is done by either family physicians or pediatricians, depending on organizational assistance, presence of chronic disease, or patient or parental preference.

Although cost studies have been carried out in other European countries, information on health costs related to JIA is scarce in Spain. Medication and healthcare costs in childhood are covered by Spain’s national health system, but other burdens associated with the disease directly impact the family of the child with JIA, such as loss of productivity, family care, or trips to the hospital. A deeper understanding of the overall burden of JIA could help stakeholders design health strategies better adapted to the needs of the environment of children with JIA. Therefore, the objective of this study was to evaluate the direct healthcare, indirect resource utilization, and related costs of moderate-to-severe JIA in standard clinical practice in Spain.

## METHODS

The ITACA OR (Outcomes Resources in Cohort from Immunogenicity and Treatment Efficacy in Children with JIA Treated with Anti-TNF and Calprotectin as Disease Activity Biomarker; multicentric study) is a 24-month retrospective study of children who had previously participated in the ITACA study (PFI-ETA-2013-01) from 2016 to 2018.^26^ The ITACA study was a 12-month observational and prospective study that included outpatients of both genders younger than 18 years diagnosed with nonsystemic JIA according to the International League of Associations for Rheumatology criteria (JIA can be diagnosed if age at onset is <16 years, disease duration is ≥6 weeks, and other known conditions are excluded) and previously treated with TNF-α treatment (inclusion criteria of ITACA cohort).

Written informed consent was taken from the parents or legal tutor of all enrolled children. In addition, children over 7 years of age provided assent. In accordance with the Spanish recommendations, this 24-month retrospective study was approved by the Clinical Research Ethics Committee of La Fe Hospital apart from the 12-month ITACA study and was conducted following the principles included in the Declaration of Helsinki for studies in human subjects.

The study was conducted among children attending the pediatric rheumatology department in 3 tertiary-care hospitals in Spain in the context of a single routine visit. Data were recorded retrospectively over a 24-month period. A structured questionnaire was designed to include national health system resource use, family of patient out-of-pocket expenses, and data regarding parent work impact (**Supplementary File 1**).

Data collection included demographics and clinical characteristics of children (age, sex, place of residence, disease duration) and use of healthcare resources (treatments, visits with the specialist physicians, visits to the emergency department, hospitalizations, surgeries, laboratory and other tests, medical devices, and physiotherapy). Parents completed a structured questionnaire, designed to evaluate annual direct and indirect costs, and were interviewed regarding costs related to the disease in the last 24 months (number of days/hours missed due to JIA, daily hours of informal care [eg, time spent helping children with their basic daily activities], daily hours of professional care, cost of hospital visits [eg, transportation, parking, accommodations], home adaptations during the last year due to JIA, assistance and mobility devices [eg, splints, arch supports, wheelchairs, adapted chairs, walking sticks/crutches], physiotherapy, psychotherapy, and other costs). Additionally, parents and children (depending on age) completed the Childhood Health Assessment Questionnaire (CHAQ) and the Pediatric Quality of Life Inventory™ (PedsQL™). The Juvenile Arthritis Disease Activity Score (JADAS-71) was calculated from children/parent’s well-being based on a visual analog scale (VAS), joint count, erythrocyte sedimentation rate (ESR), and physician disease activity based on VAS.

The questionnaire also collected data to calculate direct costs from the National Health System. Clinical and health resource use data were obtained from a healthcare registry of electronic health records, which includes visit records (eg, medical, nursing, public physiotherapy) and complementary testing (eg, analytic and imaging tests) and parent interviews using a structured questionnaire administered by investigators (**Supplementary File S1**).

Annual direct costs were calculated based on the reported use of drugs and healthcare services and resources, using average unit prices in 2020.^26,27^

Indirect costs were estimated from the number of working days lost, using the human capital approach. Unitary costs were acquired from official sources (in 2020 euros).^27,28^ Results were reported as mean cost or resource use per patient per year. Indirect costs were calculated from the unit cost in the patient’s Case Report Form (CRF) and the total number of units consumed (Cost per Resource = Total Amount of Resource × Unit Cost), and the adjusted cost per patient [(Total Amount of Resource/Number of Patients in Study) × Unit Cost].

The JADAS-71 is used to measure JIA activity status.^29^ The JADAS-71 score is estimated as the simple linear sum of the scores from 4 components to obtain a global score of 0 to 101. These 4 components are a global assessment of disease activity by the physician and measured using a VAS of 10 cm, where 0 = no activity and 10 = maximum activity; parent/patient global assessment of well-being, measured using a VAS scale of 10 where 0 = very good and 10 = very bad; number of joints with active disease, ranging from 0 to 71; and ESR (0-10 mm/hr). In polyarticular JIA, a JADAS-71 score less than 10.5 represents high disease activity, and a score of 3.9 to 10.5 represents moderate disease activity. In oligoarthritis, high disease activity is considered to be present with a JADAS-71 score greater than 4.2, moderate with a score from greater than 2 to 4.2, and low disease activity with a score greater than 1 to 2. A JADAS-71 score under 1 reflects inactive disease for both groups.^30^

The CHAQ includes indexes of disability and discomfort.^31^ The disability index measures the functional capacity in 8 components of daily life: dressing, grooming, getting up, eating, walking, bathing, reaching, and gripping. Three components were associated with each area: (1) the extent to which daily functions are difficult to perform; (2) use of special devices; and (3) activities requiring another person’s assistance. Each question included a 4-level scale, from 0 (no difficulty), 1 (some difficulty), 2 (a lot of difficulty), to 3 (unable to perform). The nonapplicable category was added for those elements which did not apply due to the child’s age. The discomfort index assessed the estimated pain and general well-being using 2 VAS of 0 to 100 mm: a pain VAS and a well-being VAS. Higher scores were related to more severe disease activity.

The PedsQL™ questionnaire^32^ used to evaluate the health-related quality of life (HRQoL) consists of 23 elements: physical functioning (8 items), emotional functioning (5 items), social functioning (5 items), and school functioning (5 items). PedsQL™ scales comprise parallel formats of delegated reports for parents and self-reports for children. The children’s self-report includes ages 5 to 7, 8 to 12, and 13 to 18 years. The parents’ delegated report includes ages 2 to 4 (toddlers), 5 to 7 (young children), 8 to 12 (children), and 13 to 18 years (teens) and assesses the parents’ perceptions of their child’s HRQoL. Higher scores correspond to a better HRQoL.

### Statistical Methodology

A descriptive statistical analysis, including central tendency and dispersion measures for continuous variables and absolute and relative frequencies for categorical variables, was performed. Student’s *t*-test, Mann-Whitney U test, or Kruskal-Wallis H test were used to compare quantitative variables. Tests were 2-tailed with a significance level of 5%. Data were calculated using SPSS v18.0 statistical software.

## RESULTS

A total of 107 children from 3 academic hospitals were included in the study and evaluated. Most of the children (69.2%) were female with a mean (SD) age of 12.9 (4.1) years, a mean disease duration of 9.1 (3.4) years, and a mean time from diagnosis to start of anti-TNF-α treatment of 2.9 (2.8) years. According to JADAS-71, 75 (71.4%) children had inactive disease, 12 (11.4%) had low disease activity, 10 (9.5%) had moderate disease activity, and 8 (7.6%) had high disease activity. Overall, children presented with inactive/low disease activity (mean [SD] JADAS-71 scores: oligoarthritis, 1.4 [2.9]; polyarthritis 1.0 [1.9]) and a very low functional disability (mean [SD] CHAQ score 0.2 [0.4]). Children and parents referred a high HRQoL measured by PedsQL™ (**Table 1**). Of the total number of children, 37 had had uveitis prior to the study period. During the study period, 27 (25.2%) suffered at least 1 episode of uveitis, being the first episode of uveitis for 5 (5/27; 18.5%) of them. Patient characteristics data are shown in **Table 1**.

**Table 1.** Demographic and Clinical JIA Patient Characteristics (N = 107)

	**n (%)**	**Mean (SD)**	**Minimum**	**Maximum**
Age (y)		12.9 (4.1)	5.0	21.0
Female sex	74 (69.2)			
Residence urban	88 (82.2)			
Disease duration		9.1 (3.4)	4.0	19.0
ILAR classification, n (%)				
Persistent oligoarthritis	36 (33.3)			
Extended oligoarthritis	15 (14.0)			
Polyarthritis RF (+)	1 (0.9)			
Polyarthritis RF (−)	35 (32.7)			
Psoriatic arthritis	3 (2.8)			
Arthritis related to enthesitis	13 (12.1)			
Undifferentiated arthritis	4 (3.7)			
History of uveitis, n (%)	37 (34.6)			
Episodes of uveitis in the last 24 months, n (%)	27 (25.2)			
JADAS-71 (mean, SD)				
Oligoarticular		1.4 (2.9)	0.0	17.0
Polyarticular		1.0 (1.9)	0.0	8.2
JADAS-71 components				
Active joints		0.1 (0.4)	0	2
Patients with:				
0 active joints	95 (88.8)			
1 active joint	10 (9.3)			
2 active joints	2 (1.9)			
ESR		11.9 (15.7)	1	100
Medical disease assessment (VAS)		0.3 (0.9)	0	5.7
Parent/patient functionality assessment (VAS)		0.5 (1.4)	0	8.2
JADAS-71^a^				
Inactive	75 (71.4)			
Low disease activity	12 (11.4)			
Moderate disease activity	10 (9.5)			
High disease activity	8 (7,6)			
CHAQ^b^		0.2 (0.4)	0.0	2.0
Mild dysfunction	99 (92.5)			
Moderate dysfunction	6 (5.6)			
PedsQL^TM^ (0-100), mean (SD)				
Children		84.8 (15.2)	37.0	100.0
Parents		82.0 (17.4)	31.5	100.0

^a^JADAS-71 cut-off for oligoarthritis and polyarthritis: inactive, ≤1 (both); low, 1-2 and 1-3.8; moderate, >2-4.2 and >3.8-10.5; high, >4.2 and >10.5, respectively.

^b^CHAQ cut-off: mild, ≤1; moderate, 1 to ≤2.

Abbreviations: CHAQ, Childhood Health Assessment Questionnaire; ESR, erythrocyte sedimentation rate; ILAR, International League of Associations for Rheumatology; JADAS-71, Juvenile Arthritis Disease Activity Score; JIA, juvenile idiopathic arthritis; PedsQL™, Pediatric Quality of Life Inventory; VAS, visual analog scale.

The pattern of utilization of direct healthcare resources is represented in **Table 2**. Almost all the children (94.4%) received some JIA treatment. Most of them (96.0%) were still on one bDMARD; in particular, 46 (47.4%) children received adalimumab and 39 (40.2%) etanercept. Fifty-four (53.5%) children received MTX. Sixteen (15.0%) children needed additional uveitis treatment. Medical visits were another important expense, especially in pediatric rheumatology, but also visits to general pediatrics and traumatology, ophthalmology and surgery (**Table 2**). During the study period, 15 patients (14.4%) required surgical treatment; in 6 of them, surgery was related to JIA. The mean (SD) number of surgeries was 1 (0.0) during the 24 months of analysis. All patients required laboratory testing (eg, blood cell counts, ESR, C-reactive protein, and liver and renal function) during the study period. The mean (SD) number of medical tests was 13.1 (7.6) during the 24 months of analysis. Only 5 children received physiotherapy (20 sessions in total for 5 patients) from the National Health System. Outside the National Health System, 11 children received 24 physiotherapy sessions, which are included in the costs assumed by the family.

**Table 2.** Utilization Pattern of Direct Healthcare Resources in 24 Months

	**n (%)**	**Minimum**	**Maximum**
**Treatments**	101 (94.4)		
Biologic DMARD (n, %)	97 (96.0)		
Monotherapy (n, %)	82 (84.5)		
In combination with csDMARD	15 (15.5)		
MTX	54 (53.5)		
Other csDMARD	15 (14.9)		
Corticosteroids	3 (3.0)		
Adalimumab	46 (47.4)		
Etanercept	39 (40.2)		
Tocilizumab	9 (9.3)		
Abatacept	1 (1.0)		
Infliximab	1 (1.0)		
Secukinumab	1 (1.0)		
**Specialist visits (N = 107)**			
Primary healthcare pediatrics	101/107		
No. of visits, mean (SD)	11.6 (12.3)	0.0	88.0
Pediatric rheumatology	107/107		
No. of visits, mean (SD)	11.2 (6.1)	4.0	39.0
Other specialists	107/107		
No. of visits, mean (SD)	9.0 (8.7)	0.0	52.0
Day hospital	106/106		
No. of visits, mean (SD)	1.8 (6.0)	0.0	35.0
Emergency department	1/102		
No. of visits, mean (SD)	1.5 (2.1)	0.0	11.0
Hospital admissions	12/106		
Length of stay (days), mean (SD)	0.5 (1.6)	0.0	9.0
Surgeries	15/107		
No. of surgeries, mean (SD)	1.0 (0.0)	1.0	1.0
Medical test	107/107		
No. of medical tests, mean (SD)	13.1 (7.6)	1.0	49.0
Public physiotherapy	5 /102		
No. of sessions, mean (SD)	0.2 (1.4)	0.0	14.0

Abbreviations: csDMARD, conventional synthetic disease-modifying antirheumatic drugs; MTX, methotrexate.

Over 24 months of observation, 64% of the fathers and 68.9% of the mothers had work absences due to child’s JIA; fathers missed a mean (SD) of 6.9 (6.0) days, whereas mothers missed a mean of 8.0 (5.8) days (**Table 3**). Among other indirect resources, 83.2% of the parents used their own vehicle to attend visits. Further, of the 14 children who were hospitalized due to JIA, 23.4% of parents needed accommodation during hospitalizations with a mean of 3.7 nights (SD, 7.2). Overall, 10.3% of the children required assistance and mobility devices, 12.3% required private physiotherapy, and 34.3% required private psychology support. Sixteen parents (15%) also required private psychological support (**Table 3**).

**Table 3.** Utilization Pattern of Indirect Resources

	**n (%)**	**Minimum**	**Maximum**
JIA impact on parents’ work absences			
Father’s work absence days due to child’s JIA (N = 100)			
n (%)	64 (64.0)		
Work absences in days, mean (SD)	6.9 (6)	0.0	28.0
Mother’s work absence days due to child’s JIA (N = 101)			
n (%)	71 (68.9)	0.0	30.0
Work absences in days, mean (SD)	8.0 (5.8)		
Other indirect healthcare resources			
Auto transportation (N = 107)			
n (%)	89 (83.2)		
Professional care for other child(ren) during hospital visit (N = 102)			
Babysitter, n (%)	13 (12.1)		
Grandparents, n (%)	7 (6.5)		
Family accommodation during hospitalizations due JIA (N = 104)			
Yes, n (%)	25 (23.4)		
Assistance/mobility devices (N = 107)			
Yes, n (%)	11(10.3)		
Physiotherapy (N = 88)			
Yes, n (%)	11 (12.5)		
Psychological support (children)			
Yes, n (%)	23 (34.3)		
Psychological support (parents) (N = 62)			
Yes, n (%)	16 (15.0)		

Abbreviation: N, number of patients answering the question.

Total mean (SD) costs caused by JIA during a 12-month period were €7516.40 (€5627.30). The mean (SD) annual cost of drug treatment was €3021.60 (€3628.90); the main drug expenses were related to biologic therapy. The mean (SD) direct annual cost (excluding treatments) was estimated at €3747.60 (€3956.20). The highest costs were due to treatment administration costs and visits to pediatric rheumatologists and primary care pediatricians (**Table 4**). JIA annual indirect expenses amounted to €747.20 (SD, €1452.80). The highest costs were generated by parents’ absences from work, babysitting expenses, and transportation (mainly by car). Regarding indirect healthcare and non-healthcare costs per year, expenditures due to the parents’ work absences constituted one of the largest shares for all indirect costs: €148.30 (SD, €254.10) for fathers and €183.70 (SD, €279.60) for mothers. The mean annual expenditures per child was €167.50 (SD, €1139.30) for childcare, €5.60 (SD €26.50) for assistance and mobility devices, €15.10 (SD, €78.30) for private physiotherapy, and €7.01 (SD, €35.30) for accommodations during hospitalizations due to JIA.

**Table 4.** Annual JIA Cost per Patient (N = 107)

	**Cost (€), Mean (SD)**	**Minimum**	**Maximum**
Total JIA cost	7516.40 (5627.30)	832.80	32 089.90
Treatment JIA cost	3021.60 (3628.90)	0.0	16 255.80
Biological DMARDS	2789.00 (3399.80)	0.0	16 128.00
MTX	10.90 (24.40)	0.0	127.80
csDMARD	220.60 (973.50)	0.0	7300.00
Oral corticosteroids	0.20 (1.40)	0.0	10.80
NSAID	0.10 (1.50)	0.0	15.00
Uveitis treatment^a^	0.80 (4.30)	0.0	40.90
Other direct costs	3654.60 (3899.00)		
Treatment administration	757.80 (2616.40)	0.0	21 715.90
Primary healthcare pediatrics	555.30 (641.40)	0.0	4545.10
Pediatric rheumatology	649.20 (364.50)	205.90	2193.40
Other specialists	524.70 (565.80)	0.0	4040.70
Hospitalizations	192.60 (659.10)	0.0	3872.80
Emergency	111.70 (163.50)	0.0	768.90
Surgery	234.10 (1099.20)	0.0	10 207.10
Tests	520.80 (430.30)	11.20	2518.50
Physiotherapy (public healthcare system)	2.10 (15.40)	0.0	155.10
Nursing	13.40 (138.10)	0.0	1428.70
JIA indirect costs	747.20 (1452.80)	24.7	14 322.90
Father’s work absences	148.30 (254.10)	0.0	1507.50
Mother’s work absences	183.70 (279.60)	0.0	1938.20
Professional care (babysitter)	167.50 (1139.30)	0.0	11 550.00
Car transportation cost	120.80 (124.40)	0.0	630.00
Other	126.90		

^a^Other than DMARD treatment.

Abbreviations: csDMARD, conventional synthetic disease-modifying anti-rheumatic drugs; JIA, juvenile idiopathic arthritis; MTX, methotrexate; NSAID, nonsteroidal anti-inflammatory drugs.

Some resource consumption (direct and indirect) showed great variability among the children, which is reflected in the annual cost; the maximums and minimums are reflected in **Tables 2, 3,** and **4**.

A comparison was made between costs associated with disease activity. According to JADAS-71, 70% of patients had inactive disease, and the groups with moderate and high disease activity were relatively small; therefore, the comparison was made between combining patients with low, moderate, and high disease activity in the same group (30%). There were no statistically significant differences between the group of patients with inactive disease and the group of patients with disease activity (low, moderate, or high) with respect to the total annualized expenditure (*t* = 0.167; *p* = .869). Descriptively, the lowest mean corresponded to the group of inactive disease (€7086.40) compared with the group of low, moderate, or high activity (mean, €7269.40). When the cost items were analyzed independently, there were also no statistically significant differences between the group of patients with inactive disease and the group of patients with low, moderate, or high disease activity with respect to annualized JIA treatment expenditure (*t* = 0.125; *p* = .900), annualized direct treatment expenditure (excluding main treatment) (*t* = 0.341; *p* = .734), or annualized indirect expenses (*t* = 0.108; *p* = .914).

## DISCUSSION

The present study is the first to provide descriptive data on the cost and pattern of use of healthcare and non-healthcare resources in a cohort of children with moderate to severe JIA in Spain in daily clinical practice. Overall, children had been receiving long-term biologic treatment and presented sustained inactive/low disease activity and very low functional disability. We calculated the total costs caused by JIA during a 12-month period at €7516.40 (SD, €5627.30). Expenditures on medicines accounted for the largest share of total healthcare costs. Mean cost of medication was €3021.80 (SD, €3628.90). Main charges for pharmacological treatment were due to biologic therapy with a mean annual cost of €2789.00 (SD, €3399.80). Non-treatment-related healthcare costs were estimated at €3747.60 (SD, €3956.20). Indirect costs of JIA per family reached €747.20 (SD, €1452.80).

Several studies have reported greater costs than those showed herein. In a current cross-sectional study performed in 6 European countries, Kuhlmann et al^24^ reported an annual cost from 125 pediatric JIA patients ranging from €18 913 per patient (France, 2012) to €45 227 (Sweden, 2012). The estimated direct healthcare cost ranged from €11 068 per patient (France, 2012) to €26 985 in (Sweden, 2012), while the direct non-healthcare cost ranged from €7837 per patient (Italy, 2012) to €18 242 (Sweden, 2012).^24^

A recent study carried out in 3815 patients with JIA who initiated biologic and non-biologic treatment showed a mean of total unadjusted all-cause healthcare costs of €18 611 (SD, €42 104) (US, 2008-2016).^33^

In concordance with our results, other authors have reported that the greatest contribution to costs was due to medication, ranging from 50% to 90% of the total healthcare cost.^12,18,34^ Data regarding the cost of anti-TNF-α drugs in JIA are very scarce.^12,14,18,24^ Although anti-TNF-α treatments have proven efficacious in the treatment of JIA and are included in treatment guidelines,^35^ the increase in costs and the lack of long-term cost-effectiveness data contribute to reasons why they are usually not prescribed earliest in the disease course.^14^ Ungar et al carried out a cost-effectiveness analysis using data from a systematic review.^21^ These authors included randomized controlled assays, observational studies, and systematic reviews designed to evaluate the cost of anti-TNF drugs for the treatment of JIA, polyarticular arthritis, or juvenile rheumatoid arthritis.^21^ Data showed that the annual total cost of a treatment could range from $13 748 to $17 981 and the annual total cost from $16 608 to $18 966 (Canada, 2008),^21^ depending on the anti-TNF treatment.

Data regarding cost of csDMARD vs bDMARD are even more limited. A prospective study in Finland compared the cost during the first year of JIA^36^ of 3 treatment options: infliximab (IFX), csDMARD, and MTX alone. Using prices for biosimilar IFX in the short-term, biosimilar IFX + MTX can be considered cost-effective when compared with MTX alone with a cost of treatment adjusted annual mean (SD): IFX + MTX, €21 164 (€4158); MTX, €18 300 (€8635), and csDMARD, €12 136 (€5286). However, compared with MTX monotherapy, biosimilar IFX + MTX could be considered cost-effective at the commonly accepted willingness-to-pay level of £30 000/QALY. As csDMARD showed good efficacy at a reasonable cost, it should not be overlooked as a therapeutic option in JIA. Since all anti-TNFs, IFX in children, and csDMARD are not the most used treatments in our clinical practice, it is difficult to establish comparisons with the ITACA study; in addition, the JIA duration in the patients included in the Finnish study was 1 year, while, in our study, the disease duration (mean, SD) was 9.1 (3.4) years.

In the last 5 years, the introduction of biosimilars have reduced the costs of therapy, such as the original bDMARDs, in most European countries, including Spain. This could represent a reduction of the main direct cost and therefore a better cost-effectiveness profile.

In previous studies, direct costs were highest among patients with more severe disease and greater disability.^33,37,38^ In this study, we found a large majority of children with JIA in low disease activity (LDA or remission Therefore, with a good treatment response, in which anti-TNF treatment was provided early in the disease and long-term bDMARD treatment was maintained, there is a higher rate of a good quality of life and a very low functional disability state, showing a reduced direct healthcare cost. Despite this, JIA still has a social, occupational, and economic resource consumption impact on parents of a child with this condition.

Recent data have demonstrated that anti-TNF drugs reduce direct and indirect healthcare costs when the drug cost itself is excluded.^12,19^ Furthermore, better control of JIA may lead to much improved long-term clinical results, which could affect social function, work, quality of life, and healthcare expenses.^12,21,39,40^ In addition, other studies show that biologic treatment can decrease the long-term complications and social burden of JIA.^14,33,41^ In a follow-up study of adult patients diagnosed with JIA in childhood, those in remission had healthcare costs 7 times lower than those with active disease.^33,39^

In addition to the direct costs, we cannot ignore the indirect costs, which are mainly borne by families. In cost analysis, indirect costs of JIA are underrepresented.^42^ It has been estimated that a mean out-of-pocket cost of €223 ranged from 15% for school costs to 50% for transportation costs.^43^ Another element that may be omitted in economic evaluations of JIA concerns the use of complementary health care. This kind of health care is used by a considerable proportion of children with JIA and forms part of families’ out-of-pocket costs.^43^ Herein, indirect JIA costs per family amounted to €747.20 (SD €1452.80), which included, in addition to loss of labor productivity, expenditures per patient for assistance and mobility devices, private physiotherapy, professional care, accommodations during hospitalizations, and transportation.

Recently, one study reported an increase in absenteeism in more than half of parents during the first year after their child’s disease diagnosis.^25^ Similarly, certain authors have indicated that 20% of parents of children with JIA request a leave of absence during the first year after the child’s diagnosis, and 3% stop working in order to take care of the child.^25,44^ Minden et al reported a mean indirect cost due to time loss from work of €270 per year per German family in 2009.^37^ Our results show that annual expenditures (Spain, 2020), due to the parents’ work absences represented one of the largest factors for all indirect cost: €148.30 (SD €254.10) in the case of the fathers and €183.70 (SD €279.60) for mothers.

A recent study in Spain described the characteristics, prevalence of comorbidities, and use of primary care services by chronic pediatric patients and analyzed factors associated with the weight of complexity according to adjusted morbidity groups (AMG).^45^ Patients with chronic disease under 18 years of age, stratified by risk levels, were analyzed. Primary care consultation is one of the most consumed resources, and the average number of visits to the primary care pediatrician was 4.9 (SD, 6.3). The most prevalent chronic diseases were asthma (38.8%), attention deficit hyperactivity disorder (ADHD) (1.8%), anemia (1.7%), and obesity (1.4%). Healthcare contact, in-person interactions, and pediatrician consultations per year were higher in medium-risk than in low-risk patients [23.4 (18.2)/22.9 (15.3)/18.4 (13.7) vs 7.5 (6.9)/7.8(6.2)/4.4 (5.4)] (*p* < .01). In our study, the number of consultations (mean [SD]) to primary healthcare pediatric services of JIA patients was 11.6 (12.3) in a period of 24 months, as expected in chronic pathologies classified as mild, which is consistent with the JIA status (disease activity, disability and quality of life) shown by the patients in the study, despite the fact that the patients included had moderate to severe JIA that had required bDMARD.

A possible limitation of the present study is that the results can only be generalized to a specific population of children with moderate to severe nonsystemic JIA who have required treatment with biologic DMARDs; followed up in a tertiary hospital; and had a long evolution of the disease, long-term treatment with bDMARD, and a close disease follow-up. On the other hand, there is a high standard deviation on cost values that could be related to differences between disease activity of the patients included, almost all of whom were in remission, but some of whom had high disease activity. The number of children with JIA and moderate and high activity in our study is small (9.6% and 7.6%, respectively), as with the number of patients with disability (5.6% with moderate and none with severe), which has not allowed cost comparisons between the groups or the ability to find significant differences. Other study limitations are the comparison at different periods of the disease and the time on treatment, comparison with csDMARDs treatment, and the lack of data on the cost of the patient’s route to reach remission of the disease.

In conclusion, JIA patients incur considerable healthcare costs. JIA generates important charges to the Spanish healthcare system and patients’ families. Compared with previous studies, our results show lower annual healthcare costs. Several reasons could explain this finding: centers are reference centers with high expertise in patient management, early diagnosis and treatment, close patient follow-up, and the usage of long-term biologic treatments. All these factors make it possible to achieve and maintain inactive/low disease activity and a very low functional disability and, as consequence, a reduced healthcare cost. Public costs are partially due to the high cost of biologic drugs, which have also proved to be an effective long-term treatment to maintain inactive or low disease activity, a very low functional disability, and a good quality of life. Real cost-consequence studies of treatments and patient management are necessary to introduce not only the direct cost related to the medication, but also the whole set of other direct and indirect costs, into the equation.

## Supplementary Material

Supplementary Appendix 1 List of Items Collected

Supplementary Appendix 2
