# Supplementary material for: Real-World Health Care Outcomes and Costs Among Patients With Juvenile Idiopathic Arthritis in Spain
Source: J Health Econ Outcomes Res. 2023 Dec 20;10(2):141–9. doi: 10.36469/001c.85088 (PMC10742379; doi:10.36469/001c.85088)
Supplement: Supplementary Appendix 1 — List of Items Collected [file jheor_2023_10_2_85088_181726.pdf]

## Supplementary Appendix 1. List of Items Collected

### A. CONSUMPTION OF HEALTH RESOURCES (DIRECT AND INDIRECT COSTS) DATA OBTAINED FROM STRUCTURED INTERVIEWS WITH PARENTS/CAREGIVERS OF THE PATIENT

1.- What is the relationship of the person completing this survey to the child with JIA?
- ☐ Mother
- ☐ Father
- ☐ Other (please specify _____)

2.- Residence
- ☐ Rural
- ☐ Urban

3.- Level of education (father)
- ☐ Basic
- ☐ High school
- ☐ Vocational training
- ☐ Graduate
- ☐ Bachelor or higher

4- Level of education (mother)
- ☐ Basic
- ☐ High school
- ☐ Vocational training
- ☐ Graduate
- ☐ Bachelor or higher

5.- Occupation (father)
- ☐ Full-time work
- ☐ Part-time work
- ☐ Housewife
- ☐ Retired
- ☐ Unemployed

6.- Occupation (mother)
- ☐ Full-time work
- ☐ Part-time work
- ☐ Housewife
- ☐ Retired
- ☐ Unemployed

7.- Father's profession
- ☐ Services (administrative, catering, hospitality...)
- ☐ Drivers, labourers (construction, agriculture, fishing), customer service (
- ☐ Qualified machinery operators, skilled manufacturing, skilled office clerk, skilled construction workers, safety, and security services.
- ☐ Qualified professionals and technicians,
- ☐ Executive
- ☐ Other

8.- Mother's profession
- ☐ Services (administrative, catering, hospitality…)
- ☐ Drivers, labourers (construction, agriculture, fishing), customer service (
- ☐ Qualified machinery operators, skilled manufacturing, skilled office clerk, skilled construction workers, safety, and security services.
- ☐ Qualified professionals and technicians,
- ☐ Executive
- ☐ Other

9.- Level of education (patient): ____________________

10.- Does the level of education correspond to the age of the patient?
- ☐ Yes
- ☐ No

11.- Because of JIA, how many hours/day do you spend helping child with basic day-to-day activities? _______hours

12.- Do you need a professional caregiver for your child?
- ☐ No (Skip to question 13)
- ☐ Yes
- ☐ Grandparents
- ☐ Other

If yes, how many hours per week? ______**hours** How much do you spend monthly? **__________ €**

13.- Are there other children at home who require a babysitter while you are on hospital visits for the child with JIA?
- ☐ No (Skip to question 14)
- ☐ Yes
- ☐ Grandparents
- ☐ Other

If yes, how much do you typically spend on babysitters for each hospital visit? ________€

14.- How close is your home from the hospital? _________Kilometers

15.- How long does it take to the hospital from home? _____ hours __________minutes

## Supplementary Appendix 1. List of Items Collected

16.- What means of transport do you use to attend the visit?

- ☐ Public transport (metro, bus, taxi)
- ☐ Car
- ☐ Train
- ☐ Airplane

17.- If you drive to the hospital for a visit, how much do you usually spend on fuel? Please indicate the total diesel/petrol for the round trip. ____________________€

18.- If you drive to the hospital for a visit, how much do you usually spend on parking? ______________€

19.- If you travel by another means of transport, how much does it cost? __________________________€

20.-During hospitalization due to JIA, should you ever spend the night away from home?

- ☐ No (Skip to question 21)
- ☐ Yes. If yes, how many nights have you had to spend away from home in the last 24 months? _____________ Nights out in the last year

Please indicate here how much you usually spend on your accommodation per night

| Accommodation | € Cost per night |
|---|---|
| Hotel | € Cost per night |
| Other | € Cost per night |

21.- Have you had to make accommodations at home during the past 24 months because of JIA?

- ☐ No (Skip to question 22)
- ☐ Yes. If yes, what changes did you make over the past 24 months and how much did each adaptation cost? Please indicate only the amount of direct money you have paid and has not been refunded.

| Home adaptation | Cost for adaption of house in the last year |
|---|---|
| 1. | |
| 2. | |
| 3. | |

22.- In the past 24 months, has the child needed any help because of his or her arthritis? For example, splints, orthotics, wheelchairs, modified chairs, cane/crutches, etc.

- ☐ No (skip to question 23)
- ☐ Yes. If yes, indicate the different aids that the child has used and the amount of money you have paid for each aid during the last 24 months.
- ☐

| Aid | Cost |
|---|---|
| 1. | € |
| 2. | € |
| 3. | € |

23.- How much have you spent in the last 24 months on physical therapy fees? ___________€

24.- Psychosocial support for children

- ☐ He's had it.
- ☐ You've needed it
- ☐ Nº of hours _____
- ☐ Public ☐ private funding

25.- Psychosocial support for parents

- ☐ He's had it.
- ☐ You've needed it
- ☐ Nº of hours _____
- ☐ Public ☐ private funding

26.- Do you need to stay overnight away from home for your JIA visit?

- ☐ No (Skip to question 27)
- ☐ Yes. If yes, how much do you usually spend on your accommodation per night? ____________€

27.- (Father) During the past 24 months, have you had to take any work leave to attend visits, hospitalizations, or other reasons related to the child's JIA?

- ☐ No (Skip to question 28)
- ☐ Yes. If yes, how many hours did you miss for each hospital visit? ____________hours

29. (Mother) During the past 24 months, have you had to take any work leave to attend visits, hospitalizations, or other reasons related to the child's JIA?

- ☐ No (Skip to question 30)
- ☐ Yes. If yes, how many hours did you miss for each hospital visit? _____________hours

## Supplementary Appendix 1. List of Items Collected

28.- (Father) How many workdays/hours did you lose in the last 24 months due to hospital visits and hospitalizations? _______days _____hours

31.- (Father) Has your child's illness impacted your work since you started the illness?

- ☐ Nothing
- ☐ Little
- ☐ A lot
- ☐ You have had to change jobs because of your child's illness
- ☐ You have lost your job because of your child's illness

30.- (Mother) How many workdays/hours did you lose in the last 24 months due to hospital visits and hospitalizations? _______days _____hours

_________________________days

32.- (Mother) Has your child's illness impacted your work since you started the illness?

- ☐ Nothing
- ☐ Little
- ☐ A lot
- ☐ You have had to change jobs because of your child's illness
- ☐ You have lost your job because of your child's illness

**B. CONSUMPTION OF NATIONAL HEALTH SYSTEM RESOURCES (DIRECT COSTS).**
**DATA EXTRACTED FROM THE HEALTHCARE REGISTRY OF THE NATIONAL HEALTH SYSTEM**

| | | |
|---|---|---|
| **Devices and healthcare treatment for treatment delivery (IV drugs/SC drugs)** | | |
| Number of drug administrations in the last 24 months: _____ | | |
| **Visits to specialists and Emergencies (if none type 0 in the text field)** | | |
| Number of visits to Primary Care Pediatrics | | ________ consultations |
| Number of visits to Pediatric Rheumatology | | ________ consultations |
| Number of visits to other specialists | | ________ consultations |
| Number of visits to Day Hospital | | ________ consultations |
| Number of visits to the Emergency Department | | ________ consultations |
| **Hospital admissions** | | |
| Number of hospital admissions | | ________ Hospital admissions |
| Approximate duration of hospital admissions | | ________ days |
| **Surgeries** | | |
| ○ Yes →<br>○ No<br>○ ND | Number of surgeries | Type of surgery |
| | __________ | 1. ______________________________<br>2. ______________________________<br>3. ______________________________ |
| **Medical exams and tests** | | |
| ○ Yes →<br>○ No<br>○ ND | Number of medical examinations and tests | Type of medical exam or test |
| | __________ | 1. ______________________________<br>2. ______________________________<br>3. ______________________________ |
| **Number of public physiotherapy sessions (if none type 0 in the text field)** | | _________________ Physiotherapy sessions |
