# Supplementary material for: Real-World Health Care Outcomes and Costs Among Patients With Juvenile Idiopathic Arthritis in Spain
Source: J Health Econ Outcomes Res. 2023 Dec 20;10(2):141–9. doi: 10.36469/001c.85088 (PMC10742379; doi:10.36469/001c.85088)
Supplement: Supplementary Appendix 2 [file jheor_2023_10_2_85088_190199.pdf]

Supplementary Appendix 2

Table S1. Health resources and direct cost analysis "Health Costs". Summary of the analysis of resources and costs in 24 months. The calculation of the cost of the resource on the total N of patients included in the statistical analysis of the study is included.

Health Resources (1)	Average amount of resource consumed	Total patients with consumption	Total amount of resource consumed	Average amount of resource over total N (N=107)	Unit cost	Total Cost	Cost per patient	Cost per patient adjusted for total N (N= 107)
	A	B	C	D	E	CxE	BxE	(CxE) / N
**bDMARDs treatments for JIA**	Average amount of resource (mg)	Valid N	Total quantity of resource (mg)	Total amount of resource (mg) over total N (N=107)	Cost (mg) PVL /reference PVL	Cost per resource (quantity x cost)	Cost per patient [(quantity/N valid) x cost]	Cost per patient adjusted for total N (N= 107)
Etanercept	1314.97	39	51283.93	479.29	3.38	173339.68	4444.61	1620.00
Adalimumab	1010.71	46	46492.6	434.51	7.84	364501.98	7923.96	3406.56
Tocilizumab	3497.84	9	31480.6	294.21	1.51	47535.71	5281.75	444.26
Secukinumab	803.60	1	803.6	7.51	3.81	3061.72	3061.72	28.61
Abatacept	5250.00	1	5250	49.07	1.51	7927.5	7927.5	74.09
Infliximab	117.33	1	117.33	1.10	4.02	471.67	471.67	4.41
**Total, bDMARD expenditure**						**596838.26**		**5577.93**
**csDMARD treatments for JIA**	Average amount of resource (mg)	Valid N	Total quantity of resource (mg)	Total amount of resource (mg) over total N (N=107)	Cost (mg) PVL /reference PVL	Cost per resource (quantity x cost)	Cost per patient [(quantity/N valid) x cost]	Cost per patient adjusted for total N (N= 107)
**Metotrexate**	5355.12	54	289176.7	2702.59	0.02	5783.5	107.10	54.05
**Micofenolate**	848000.00	4	3392000	31700,93	0,01	33920	8480,00	317.01
**Leflunomide**	11195.00	10	111950	1046.26	0,06	6717	671.70	62.78
**Azatioprine**	36500.00	1	36500	341.12	0.18	6570	6570.00	61.40
**Corticosteroid treatment for JIA**	Average amount of resource (mg or number of infiltrations)	Valid N	Total quantity of resource (mg or number of infiltrations)	Total amount of resource (mg or number) over total N (N=107)	Cost (mg or number) PVL /reference PVL	Cost per resource (quantity x cost)	Cost per patient [(quantity/N valid) x cost]	Cost per patient adjusted for total N (N= 107)
**Oral corticosteroids**	1448.3	3	4345	40.61	0.01	43.45	14.48	0.41
**Intraarticular corticosteroid**	1.4	5	7	0.07	0.06	0.42	0.08	0.004
**NSAIDs for JIA (regularly scheduled)**	Average amount of resource (mg)	Valid N	Total quantity of resource (mg)	Total amount of resource (mg) over total N (N=107)	Cost (mg) PVL /reference PVL	Cost per resource (quantity x cost)	Cost per patient [(quantity/N valid) x cost]	Cost per patient adjusted for total N (N= 107)
**NSAIDs (regularly scheduled)**	50.00	1	50	0.47	0.20	10.00	10.00	0.09
**Current treatments for JIA. New uveitis treatments**	Average amount of resource (mg)	Valid N	Total quantity of resource (mg)	Total amount of resource (mg) over total N (N=107)	Cost (mg) PVL /reference PVL	Cost per resource (quantity x cost)	Cost per patient [(quantity/N valid) x cost]	Cost per patient adjusted for total N (N= 107)
**Cycloplegic**	2.00	7	7	0.07	0.26	1.82	0.26	0.017
**Phenylephrine**	1.00	1	1	0.01	3.12	3.12	3.12	0.029
**Topical corticosteroid**	1.00	1	1	0.01	0.17	0.17	0.17	0.002
**Topical ocular corticosteroid**	1.00	2	2	0.02	1.60	3.20	1.6	0.030
**Dexamethasone**	1.00	12	12	0.11	1.60	19.2	1.6	0.179
**Hydrocortisone**	1.00	1	1	0.01	1.50	1.50	1.5	0.014
**Adalimumab**	1.00	1	1	0.01	7.84	7.84	7.84	0.073
**Prednisolone acetate**	1.00	12	12	0.11	0.30	3.60	0.30	0.034
**Timolol**	1.00	1	1	0.01	0.88	0.88	0.88	0.008
**Tropicamide**	1.0	1	1	0.01	0.24	0.24	0.24	0.002

Supplementary Appendix 2

Health Resources (2)	Average amount of resource consumed	Total patients with consumption	Total amount of resource consumed	Average amount of resource over total N (N=107)	Unit cost	Total Cost	Cost per patient	Cost per patient adjusted for total N (N= 107)
	A	B	C	D	E	CxE	BxE	(CxE) / N
**Devices and healthcare treatment for treatment delivery**	Average amount of resource (number of administrations)	Valid N	Total quantity of resource (number of administrations)	Total amount of resource (number of administrations) over total N (N=107)	Oblikue cost	Cost per resource (quantity x cost)	Cost per patient [(quantity/N valid) x cost]	Cost per patient adjusted for total N (N= 107)
Drug administrations in the past 24 months without intravenous treatments* (abatacept, tocilizumab, and infliximab)	1.00	93	93	0.87	41	3813.0	41.0	35.64
Drug administrations in the past 24 months with intravenous treatments* (abatacept, tocilizumab, and infliximab)	44.27	11	487	4.55	292	142204.0	12927.64	1329.00
**Visits to specialists and Emergency**	Average amount of resource (number of visits)	Valid N	Total quantity of resource (number of visits)	Total amount of resource (number of visits) over total N (N=107)	Oblikue cost	Cost per resource (quantity x cost)	Cost per patient [(quantity/N valid) x cost]	Cost per patient adjusted for total N (N= 107)
**Primary Care Pediatrics**	11.63	101	1175	10.98	134.62	158178.5	1566.12	1478.30
**Pediatric Rheumatology**	11.24	107	1203	11.24	155.41	186958.23	1747.27	1747.27
**Another specialist**	8.96	107	959	8.96	155.41	149038.19	1392.88	1392.88
**Day Care Unit**	1.76	106	187	1.75	292.10	54622.70	515.31	510.49
**Emergency**	1.49	102	152	1.42	209.75	31882.00	312.57	297.96
**Hospital admissions**	Average amount of resource (Hospital admissions. Duration (days))	Valid N	Total quantity of resource (Hospital admissions. Duration (days))	Total amount of resource (number of admissions) over total N (N=107)	Cost Oblikue per day of admission/hospital admissions	Cost per resource (quantity x cost)	Cost per patient [(quantity/N valid) x cost]	Cost per patient adjusted for total N (N= 107)
Hospital admissions	0.51	69	35	0.33	568.48	19896.8	288.36	185.95
**Surgeries**	Average amount of resource	Valid N	Total quantity of resource	Total amount of resource (number of surgeries) over total N (N=107)	Oblikue cost	Cost per resource (quantity x cost)	Cost per patient [(quantity/N valid) x cost]	Cost per patient adjusted for total N (N= 107)
**Synovectomy wide left wrist**	1.0	1	1	0.01	1088.95	1088.95	1088.95	10.18
**Membravolectomy OI**	1.0	1	1	0.01	207.06	207.06	207.06	1.94
**Tendon release**	1.0	1	1	0.01	481.32	481.32	481.32	4.50
**Adenoidal hypertrophy**	1.0	1	1	0.01	1950.37	1950.37	1950.37	18.23
**Transtympanic drains**	1.0	1	1	0.01	486.52	486.52	486.52	4.55
**Calcaneal-astragalin coalition**	1.0	1	1	0.01	1320	1320	1320	12.34
**Knee arthroscopy, meniscectomies**	1.0	1	1	0.01	960.8	960.8	960.8	8.98
**Arthrocentesis**	1.0	1	1	0.01	184.14	184.14	184.14	1.72
**Temporomandibular arthroplasty**	1.0	1	1	0.01	1801.7	1801.7	1801.7	16.84
**Temporomandibular arthroscopy**	1.0	1	1	0.01	1801.7	1801.7	1801.7	16.84
**Scheduled burn reconstruction**	1.0	1	1	0.01	20414.26	20414.26	20414.26	190.79
**Osteosynthesis material remove**	1.0	1	1	0.01	159.46	159.46	159.46	14.92
**Varicocele**	1.0	1	1	0.01	1684.09	1684.09	1684.09	15.74
**Excision ingrown toenail**	1.0	1	1	0.01	395.99	395.99	395.99	3.70
**Fracture surgery**	1.0	1	1	0.01	2655.99	2655.99	2655.99	24.82
**Appendectomy**	1.0	2	2	0.02	6497.82	12995.64	6497.82	121.45

Supplementary Appendix 2

Health Resources (3) Direct cost	Average amount of resource consumed	Total patients with consumption	Total amount of resource consumed	Average amount of resource over total N (N=107)	Unit cost	Total Cost	Cost per patient	Cost per patient adjusted for total N (N= 107)
	A	B	C	D	E	CxE	BxE	(CxE) / N
**Medical Exams and Tests**	Average amount of resource (number of visits)	Valid N	Total quantity of resource (number of visits)	Total amount of resource (number of visits) over total N (N=107)	Oblikue cost	Cost per resource (quantity x cost)	Cost per patient [(quantity/N valid) x cost]	Cost per patient adjusted for total N (N= 107)
**Pathology**	1.33	3	4	0.04	251.93	1007.72	335.91	9.42
**Arthrocentesis**	1.67	3	5	0.05	184.14	920.70	306.90	8.60
**Audiometry**	2.00	1	2	0.02	89.26	178.52	178.52	1.67
**Nasal exudate culture**	2.00	1	2	0.02	22.37	44.74	44.74	0.42
**Echography**	5.54	63	349	3.26	64.94	22664.06	359.75	211.81
**Electroencephalogram**	2.00	1	2	0.02	362.19	724.38	724.38	6.77
**Electromyogram**	1.00	1	1	0.01	177.15	177.15	177.15	1.66
**Spirometry**	3.00	1	3	0.03	28.01	84.03	84.03	0.79
**Bone mineral densitometry**	1.67	3	5	0.05	42.82	214.10	71.37	2.00
**Blood culture**	3.00	1	3	0.03	26.09	78.27	78.27	0.73
**Impedancemetry**	3.00	1	3	0.03	84.64	253.92	253.92	2.37
**Infiltration**	3.00	1	3	0.03	231.38	694.14	694.14	6.49
**Microbiology**	3.00	1	3	0.03	26.04	78.12	78.12	0.73
**Orthopantomography**	2.50	2	5	0.05	34.7	173.50	86.75	1.62
**Allergy test**	3.00	1	3	0.03	50.7	152.10	152.10	1.42
**Imaging test**	4.00	2	8	0.07	181.31	1450.48	725.24	13.56
**Laboratory tests: Blood and urine test**	7.20	106	763	7.13	22.3	17014.90	160.52	159.02
**Breathing tests**	6.00	1	6	0.06	75.81	454.86	454.86	4.25
**Radiography**	2.48	40	99	0.93	26.39	2612.61	65.32	24.42
**MRI**	2.74	42	115	1.07	478.76	55057.40	1310.89	514.56
**Telemetry**	4.00	1	4	0.04	35.08	140.32	140.32	1.31
**Tomography**	3.75	4	15	0.14	474.98	7124.70	1781.18	66.59
**Surgery assessment**	1.00	1	1	0.01	155.41	155.41	155.41	1.45
**Physiotherapy**	Average amount of resource (number of sessions)	Valid N	Total quantity of resource (number of sessions)	Total amount of resource (number of sessions) over total N (N=107)	Oblikue cost	Cost per resource (quantity x cost)	Cost per patient [(quantity/N valid) x cost]	Cost per patient adjusted for total N (N= 107)
**Physiotherapy**	0.2	102	20	0.19	30.70	614	6.02	5.74

Supplementary Appendix 2

Table S2. Non-healthcare resources and analysis of resource use and "out-of-pocket costs for the patient". Summary of the analysis of resources and costs in 24 months. The calculation of the cost of the resource on the total N of patients included in the statistical analysis of the study is included.

Resources	Average amount of resource consumed	Total patients with consumption	Total amount of resource consumed	Average amount of resource over total N (N=107)	Unit cost	Total Cost	Cost per patient	Cost per patient adjusted for total N (N= 107)
	A = C/B	B	C	D = C/107	E			
**Time spent helping patients perform their basic day-to-day activities (hours)**	0.66	97	64.0	0.60	-	-	-	-
**Need for a professional caregiver for the child (Monthly cost €)**	12	1	12	0.11	300	7200	7200	67.29
**Need for professional care (babysitter) for other children while parents are on hospital visits for the child with JIA**	102.90	10	1029.00	9.62	-	35848.75	3584.88	335.04
**Cost of each hospital visit**								
Approximate distance from home to hospital (km)	85.12	103	8767.50	81.94	-	-	-	-
Time spent going home to the hospital (hours)	1.30	107	139.50	1.30	-	-	-	-
**Type of transport used to attend the visit**								
Public transport (metro, bus, taxi). Approximate expenditure (€)	9.61	21	201.75	1.89	-	2727.30	129.87	25.49
Car. Approximate cost on fuel + parking (€)	28.84	87	2509.40	23.45	-	25842.50	297.04	241.52
Train. Approximate expenditure (€)	41.55	12	498.60	4.66	-	4473.80	372.82	41.81
Airplane. Approximate expenditure (€)	-	-	-	-	-	-	-	-
**Need for accommodation during hospitalization due to JIA**								
Number of nights away from home in the past 24 months	3.70	20	74.00	0.69	-	-	-	-
Approximate cost of accommodation per night (€)	43.3	6	-	-	-	1500	250.00	14.02
**Domestic adaptations in the past 24 months due to JIA**	-	-	-	-	-	-	-	-
**Assistive and mobility devices for the child in the last 24 months (splints, orthotics, wheelchairs, modified chairs, cane/crutches, etc.)**								
**Contact lens**	-	1	-	-	-	450	450	4.21
**Jaw splint**	-	1	-	-	-	150	150	1.40
**Splint**	-	1	-	-	-	120	120	1.12
**Plantar orthosis**	-	1	-	-	-	150	150	1.40
**Insoles**	-	1	-	-	-	120	120	1.12
**Crutches**	-	6	-	-	-	115	19.17	1.07
**Wheelchair**	-	2	-	-	-	0	0	0.00
**Kneepad**	-	1	-	-	-	11.45	11.45	0.11
**Wristband**	-	1	-	-	-	45	45	0.42
**Total devices**	1.66	9	15	0.14	-	1191.45	132.38	11.14
**Need for private physiotherapy sessions**								
Approximate expenditure in the last 24 months on physiotherapy fees (€)	24.00	11	264	2.47	-	3250	295.45	30.37
**Need for psychosocial support**								
Number of hours (children)	19.67	3	59	0.55	-	-	-	-
Number of hours (children). Public	8.00	1	8	0.07	-	-	-	-
Number of hours (children). Private	3.00	1	3	0.03	-	-	-	-
Number of hours (parents)	30.00	1	30	0.28	-	-	-	-
**Need for accommodation for attendance at the consultation due to the JIA**								
Accommodation for the consultation due to AIJ. Approximate expenditure on accommodation per night (€)	55.00	3	165.00	1.54	-	165	55.00	1.54

Table S3. Indirect cost-labor costs. Summary of the analysis of indirect costs-labor costs (parents) in 24 months. The calculation of the cost of the resource on the total N of patients included in the statistical analysis of the study is included.

Resources	Average amount of resource consumed	Total patients with consumption	Total amount of resource consumed	Average amount of resource over total N (N=107)	Unit cost	Total Cost	Cost per patient	Cost per patient adjusted for total N (N= 107)
	A = C/B	B	C	D = C/107	E	CxE	AxE	(CxE)/107
**Father**								
Lost workdays in the last 24 months due to hospital visits or hospitalizations	3.73	100	373.10	3.49	80.31	29,963.66	299.64	280.03
Workhours lost in the last 24 months due to hospital visits or hospitalizations	23.58	100	2,358.00	22.04	22.86	53,903.88	539.04	503.77
**Mother**								
Lost workdays in the last 24 months due to hospital visits or hospitalizations	5.44	103	560.1	5.23	80.31	44,981.63	436.71	420.39
Workhours lost in the last 24 months due to hospital visits or hospitalizations	28.36	103	2,921.00	27.30	22.86	66,774.06	648.29	624.06
TOTAL	-	-	-	-	-	**195,623.23**	-	**1,828.25**
